# Diaphragm movement sensor for phrenic nerve monitoring during cryoballoon procedures: the first clinical evaluation

**DOI:** 10.3389/fcvm.2024.1361761

**Published:** 2024-03-20

**Authors:** Elsa Schemoul, Lilith Tovmassian, Julien Mancini, Linda Koutbi, Cédric Biermé, Jean-Claude Deharo, Frédéric Franceschi, Baptiste Maille

**Affiliations:** ^1^ Department of Cardiology, CHU Timone, Aix-Marseille University, Marseille, France; ^2^ Biostatistics Department, Aix-Marseille University, INSERM, IRD, ISSPAM, APHM, SESSTIM, Hôpital de la Timone, Marseille, France; ^3^ Center for CardioVascular and Nutrition Research (C2VN), INSERM, INRA, Marseille, France

**Keywords:** atrial fbrillation, cryoballoon ablation, complication, phrenic nerve—injuries, CMAP

## Abstract

**Background and aims:**

Right phrenic nerve palsy is the most frequent complication of cryoballoon procedures. The SMARTFREEZE™ console (Boston Scientific, St. Paul, MN, USA) has integrated a new tool for diaphragm monitoring—the Diaphragm Movement Sensor; however, it has not been evaluated in clinical practice. We aimed to assess the diagnostic performance of the Diaphragm Movement Sensor based on compound motor action potential data recorded simultaneously.

**Methods:**

Thirty consecutive patients (mean age 63.2 ± 10.2 years) were included. We simultaneously recorded the compound motor action potential and the Diaphragm Movement Sensor during cryoapplications in the right pulmonary veins. The right phrenic nerve was paced at 60 per minute, 12 V and 2.9 ms. Compound motor action potential monitoring with a 30% decrease cutoff for the diagnosis of phrenic nerve threatening was considered the gold standard. The Diaphragm Movement Sensor decrease threshold was also set at 30%.

**Results:**

Considering compound motor action potential monitoring, phrenic nerve threatening occurred 11 times (in seven patients) among 84 cryoapplications (13.1%) at the right pulmonary veins. The sensitivity and specificity of the Diaphragm Movement Sensor were, respectively, 33% (95% CI: 7%–70%) and 49% (95% CI: 38%–61%; *P* < 0.001). The predictive positive and negative values for the Diaphragm Movement Sensor were, respectively, 7% (95% CI: 2%–20%) and 86% (95% CI: 72%–95%). The Diaphragm Movement Sensor gave an erroneous diagnosis in 44/84 cryoapplications (52.4%).

**Conclusions:**

The diagnostic performance of the Diaphragm Movement Sensor is low, and the relevance of its use in clinical practice may be debated.

## 1 Introduction

Cryoballoon pulmonary vein isolation is a well-established, effective, and safe method (1–3) for atrial fibrillation (AF) treatment, but is associated with a significant risk of transient or permanent phrenic nerve palsy (PNP) during right-sided pulmonary vein freezes (4–6). One of the reasons may be the lack of automatic analysis of diaphragmatic contraction consecutive to right phrenic nerve pacing in the superior vena cava (7). Recently, the SMARTFREEZE™ console (Boston Scientific, St. Paul, MN, USA) has become available. A new tool for diaphragm monitoring has been integrated into this console: the Diaphragm Movement Sensor (DMS). This sensor is linked to an adhesive ECG electrode located under the right costal margin of the patient and connected to the console. Even if the algorithm is not available, it is known to be based on movement analysis. Diaphragmatic movement is expressed as a percentage. This sensor has not been evaluated in clinical practice. The statement “For reference only. Never rely solely on these indicators” is written on the console's screen. On the other hand, diaphragmatic compound motor action potential (CMAP) monitoring is used routinely in many centers (4, 8), and has been shown to be effective in preventing PNP (9).

The aim of this study was to evaluate the diagnostic performance of the DMS, using CMAP monitoring as the gold standard.

## 2 Methods

### 2.1 Study design

A prospective, single-center, comparative, observational study was undertaken. Consecutive patients admitted to the local heart rhythm department for cryoballoon ablation were included if they were aged >18 years. AF ablation was offered according to current international guidelines (2). Patients with left atrial appendage thrombus on transesophageal echocardiogram were excluded. Informed consent was obtained from the patients. The study was approved by the institutional ethics review committee.

### 2.2 Periprocedural management and cryoballoon ablation procedure

All patients underwent transesophageal and transthoracic echocardiography beforehand. Procedures were performed with uninterrupted oral anticoagulation and under deep sedation with propofol in spontaneous ventilation, associated with local anesthetic. All of the procedures were performed by a single experienced operator. A quadripolar Josephson Curve catheter (Response™; Abbott) was positioned on the His bundle, and a steerable quadripolar catheter with 4 mm electrodes and 2-5-2 spacing (Xtrem™; Microport) was placed in the coronary sinus by femoral access. These catheters were used as landmarks to perform a transseptal puncture. After vascular accesses were obtained without ultrasound guidance, a single transseptal puncture was performed to introduce an 8F transseptal nonsteerable sheath into the left atrium (SL0™; Abbott). Thereafter, patients received a weight-adjusted dose of intravenous heparin 100 IU/kg with a target activated clotting time of >300 s. The nonsteerable transseptal sheath was exchanged over a guidewire for the 15.5F POLARSHEATH™ (Boston Scientific), continuously flushed with heparinized saline solution. We only used the 28 mm cryoballoon, as the POLARx FIT™ (Boston Scientific) was not yet available. Before freezing, each vein was first cannulated with the mapping catheter (POLARMAP™; Boston Scientific). After the inflation and positioning of the balloon, complete occlusion of the vein was assessed with contrast injection. To improve the detection of pulmonary vein signals, the mapping catheter was pulled as close as possible to the tip of the balloon. The targeted freeze temperature was below −50°C. Total freezing time was set to 180 s for each application, with a target time to isolation of <60 s. When the time to isolation was >60 s, the freezing cycle was aborted and the balloon was repositioned, in order to obtain a time to isolation of <60 s. When no pulmonary vein potential could be recorded during cryoapplication, after cryoapplication, pulmonary vein isolation was assessed using the POLARMAP™. No extra freezes were routinely applied if definite isolation was achieved.

In case of cryoapplication interruption before 120 s as a result of phrenic nerve threatening assessed by a CMAP amplitude decrease >30%, a second cryoapplication was performed after CMAP amplitude recovery (full recovery or 2 min waiting time).

### 2.3 Phrenic nerve monitoring

Phrenic nerve monitoring was performed simultaneously with CMAP and DMS. CMAP was considered the gold standard and guided the physician to stop the cryoapplication in case of phrenic nerve threatening. CMAP monitoring was performed using hepatic recording. The quadripolar Josephson Curve catheter was positioned into a subdiaphragmatic hepatic vein with the aim of obtaining a CMAP amplitude >0.3 mV. Bipolar electromyographic signals were recorded between the proximal and distal electrodes. Signals were amplified and band-pass filtered between 5 and 150 Hz. After left-sided vein freezes, the steerable quadripolar catheter was relocated from the coronary sinus to the superior vena cava, to pace the right phrenic nerve at 60 stimulations per minute (bipolar stimulation between proximal and distal electrodes with maximal output of 12 V at 2.9 ms). Real-time CMAP monitoring was performed during the right-sided freezes. Phrenic nerve threatening was considered if two consecutive CMAP values were under the 30% decrease threshold. If a phrenic nerve threat was observed on hepatic signal, the application was stopped immediately using a forced deflation maneuver. Unstable phrenic nerve capture was defined as a CMAP amplitude variation of >20% from beat to beat. A phrenic nerve threat was defined as a progressive amplitude drop of 30% within 10 s of onset. In case of phrenic nerve threats, pacing was prolonged by 2 min to observe CMAP recovery. A complete recovery was defined as CMAP amplitude >90% compared with baseline, 2 min after cryoapplication was interrupted.

Phrenic nerve monitoring was performed simultaneously with DMS. The DMS sensor was attached to an adhesive electrode placed under the right costal margin and connected to the SMARTFREEZE™ cryoconsole. The data indicated by the DMS were not used to guide the procedure. To avoid distorting the DMS, diaphragmatic contraction was not monitored by abdominal palpation. Patients' movements or coughing during right-sided vein cryoapplications were collected prospectively.

After the procedure, CMAP amplitude evolution was measured beat per beat on the EP workstation (CardioLab™, General Electrics) by a trained electrophysiologist. Baseline CMAP amplitude was calculated as an average of the first five beats. The 30% cutoff timing was determined to be reached when two consecutive beats were under this threshold. Simultaneous data recorded from the DMS expressed as a percentage were extracted from the cryoconsole. Then, the diagnostic performance of the DMS was calculated using CMAP as the gold standard. The DMS was considered true positive if the 30% decrease was reached within 10 s before and 5 s after the 30% reduction in CMAP.

### 2.4 Complications definition

Major complication: death, stroke/TIA, tamponade. Minor complication: arteriovenous fistula, femoral pseudoaneurysm, pericardial effusion, phrenic nerve injury.

### 2.5 Statistical analysis

Categorical variables are expressed as absolute count (percentage) and were compared using the *χ*^2^ test or Fisher's exact test. Continuous variables are expressed as mean ± standard deviation or median [interquartile range]. The sensitivity, specificity, and predictive values of DMS and their 95% confidence intervals (CIs) were estimated using *epiR* package for R (R Core Team, R Foundation for Statistical Computing, Vienna, Austria). False positive and global error rates were compared in several subgroups (defined by patients’ movement, instability, etc.) using the *χ*^2^ test or Fisher's exact test. All tests were two-sided, and statistical significance was defined as *P *< 0.05.

## 3 Results

### 3.1 Patients and procedures

Thirty patients were included between February and September 2022. Patient and procedure characteristics are summarized in Table 1. Eighty-four applications were performed at the right pulmonary veins. Pulmonary vein isolation was achieved in 100% of cases. No complication occurred.

**Table 1:** Patient (*n* = 30) and procedure characteristics.

Characteristic	
Age, year	63.2 ± 10.2
Sex (male)	22 (73.3)
BMI, kg/m**^2^**	26.7 ± 4.3
BMI > 27 kg/m**^2^**	12 (40.0)
Paroxysmal/persistent, %	66.7/33.3
EHRA 2/3/4, %	83.3/13.3/3.3
HTN/diabetes/OSA, %	33/3.3/23.3
Antiarrhythmic treatment	20 (66.7)
Underlying cardiomyopathy	14 (46.7)
Ischemic heart disease	6 (20.0)
CHADS VASC score	1.8 ± 1.5
LVEF, %	62.1 ± 10
LA volume indexed for BSA, mL/m**^2^**	40.9 ± 11.9
Total procedure time, minutes	62 [54–88]
Fluoroscopy time, minutes	9 ± 2.3

Data are reported as mean ± standard deviation, number (%) or median [interquartile range], unless otherwise indicated.

BMI, body mass index; BSA, body surface area; EHRA, European Heart Rhythm Association; HTN, arterial hypertension; LA, left atrium; LVEF, left ventricular ejection fraction; and OSA, obstructive sleep apnea.

### 3.2 Phrenic nerve monitoring with CMAP

Among the 84 applications, CMAP monitoring indicated a phrenic nerve threat 11 times (13.1%) in seven patients. At this time, the pulmonary vein was always isolated and the temperature was −57 ± 6°C. A phrenic nerve threat was more frequent at the right superior pulmonary vein compared with the right inferior pulmonary vein (20% vs. 5.1%; *P *= 0.044). CMAP amplitude recovery was observed in 8/11 cases. Cryoapplication was halted at 104.5 ± 30 s. At this time the CMAP amplitude reduction was 39.4 ± 12%. The 30% CMAP amplitude reduction threshold was reached at 98 ± 33.9 s. Thus, the mean delay between 30% CMAP amplitude reduction and interruption of the cryoapplication was 6.5 ± 4.2 s. This delay was 5.6 ± 3.2 s and 10 ± 2.6 s in complete vs. incomplete CMAP recovery applications, respectively (NS). Cryoapplication was never halted before the 30% threshold was reached. CMAP amplitude reduction was, respectively, 33.3 ± 3.1% vs. 55 ± 11.3% in patients with and without complete CMAP recovery. In these patients, chest-x-ray showed no phrenic nerve palsy the day after the procedure.

### 3.3 DMS diagnostic performance

#### 3.3.1 Overall DMS diagnostic performance

**Figure 1:**
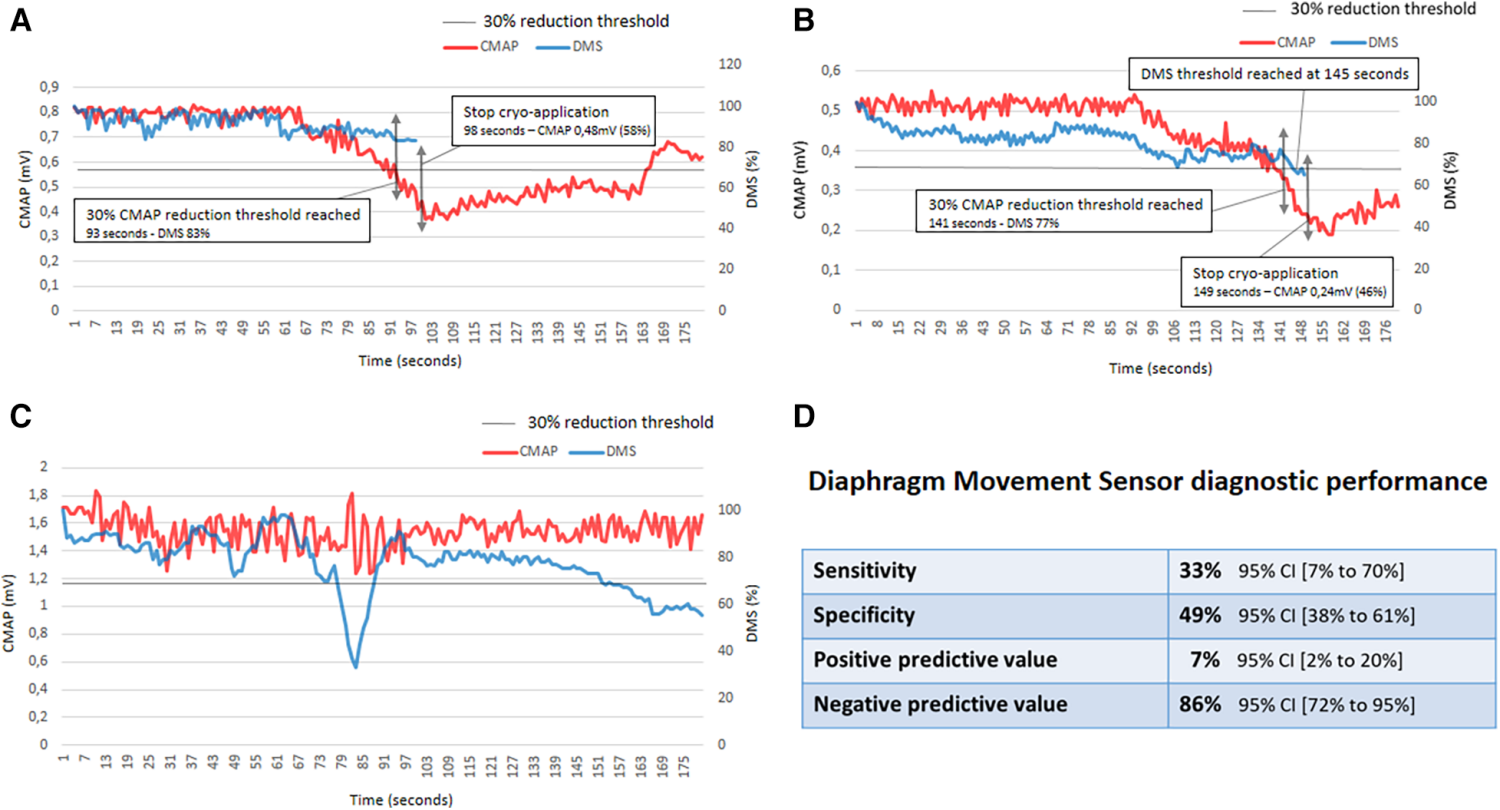
Representative figure. False negative, true positive, false positive examples and DMS diagnostic performance. (**A**) This panel represents a Diaphragm Movement Sensor (DMS) false negative. When the 30% compound motor action potential (CMAP) reduction threshold was reached, the DMS indicated a 17% reduction. The cryoapplication was stopped 5 s after the 30% CMAP reduction threshold was reached; at this time, CMAP reduction was 42%. CMAP recovery was not complete in this case. (**B**) This panel represents a DMS true positive. The 30% DMS reduction was reached 4 s after 30% CMAP reduction threshold. The cryoapplication was stopped 8 s after the 30% CMAP reduction threshold was reached. At this time, CMAP reduction was 64%. CMAP recovery was not complete in this case. (**C**) This panel represents a DMS false positive. In this case, phrenic nerve pacing was relatively unstable. As a consequence, CMAP amplitude variations were observed all along the cryoapplication. The DMS 30% reduction threshold was reached twice. (**D)** DMS diagnostic performance. CI indicates confidence interval.

DMS diagnostic performance is summarized in Figure 1. Among 84 applications, DMS false positives occurred 38 times (45.2%) in 14 patients (46.6%). It occurred 20 times in RSPV and 18 times in RIPV applications. We observed no difference in age, sex and BMI in patients with and without these false positive of the DMS. Among 11 applications with phrenic nerve threats, two DMS false positives occurred early after the beginning of the application. These two applications were ruled out for DMS sensitivity calculation. Among the nine remaining applications with phrenic nerve threats, the DMS was false negative in six (66.7%). Thus, the DMS gave an erroneous diagnosis in 44/84 cryoapplications (52.4%). No predictive factors were found for DMS false positives (sex, age, body mass index and obstructive sleep apnea were tested).

#### 3.3.2 Patients' movements during right-sided pulmonary vein cryoapplications

Despite deep sedation with propofol, movement or coughing was present in 32/84 cryoapplications (38.1%). These movements had no impact on CMAP amplitude signals and phrenic threat rate in patients with movements (5/32, 15.6%) vs. without movements (6/52, 11.5%; *P *= 0.74). On the other hand, they had an impact on the diagnostic performance of the DMS, increasing the rate of false positives. DMS specificity was 31% in patients with movements and 61% in patients without movements (*P *= 0.02).

The DMS gave an erroneous diagnosis in 65.6% of cryoapplications with patient movements and 44.2% of cryoapplications without patient movements (*P *= 0.057).

#### 3.3.3 Muscular fatigue phenomenon

**Figure 2:**
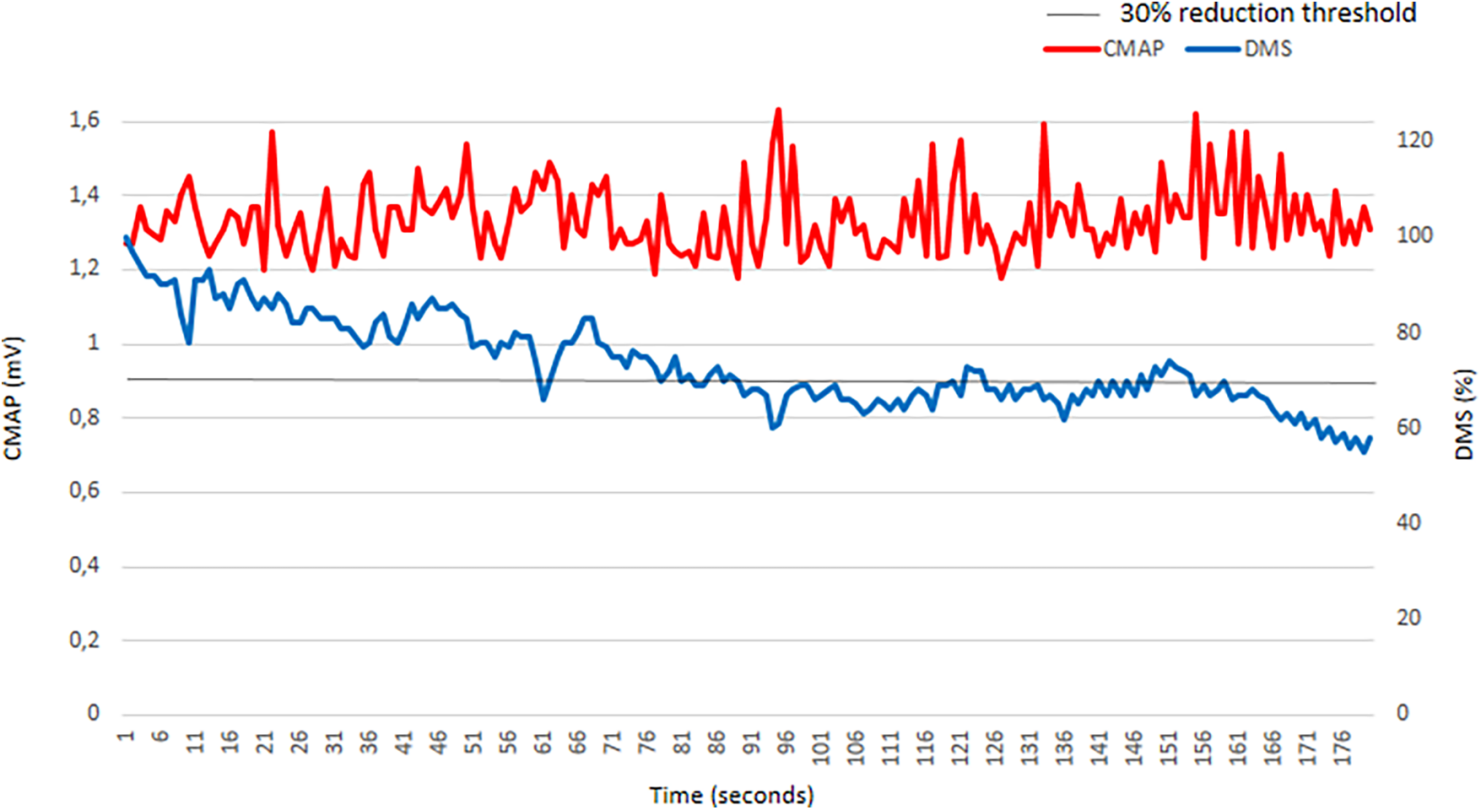
Muscular fatigue phenomenon illustration in a patient without phrenic threat. In this case, phrenic nerve pacing was relatively unstable. As a consequence, compound motor action potential (CMAP) amplitude variations were observed all along the cryoapplication. The Diaphragm Movement Sensor (DMS) value progressively declined from the beginning of the cryoapplication and reached the 30% cutoff value several times. By comparison, the CMAP value remained relatively stable all along the application. This is a DMS false positive.

In 10/84 cryoapplications (11.9%), we found a progressive decrease in the DMS value from the beginning of the application, crossing the 30% decrease threshold (Figure 2). At the same time, there were no significant variations in CMAP amplitude. This phenomenon could represents a diaphragmatic fatigue phenomenon, which led to DMS false positives. No predictive factor for this fatigue phenomenon was found.

## 4 discussion

### 4.1 DMS diagnostic performance

This study found that the DMS has a sensitivity of 33% and a specificity of 49% for the diagnosis of phrenic nerve threatening, based on CMAP monitoring as the gold standard. The DMS gave an erroneous diagnosis in 52.4% of cryoapplications. Power at al. expressed the fact that for a test to be useful, sensitivity + specificity should be at least 1.5 (halfway between 1, which is useless, and 2, which is perfect). Here, sensitivity + specificity were 0.82—far from 1.5 (10). The low specificity could lead to inappropriate interruption of cryoapplications in approximately 50% of cases, while the phrenic nerve is not threatened; this could lead to an increase in procedure and fluoroscopy time, and increase the amount of contrast used. The low sensitivity value with a high risk of false negatives exposes the patient to an adverse event (ie, it could increase the risk of PNP). Thus, relying on the DMS could be dangerous for the patient.

The diagnostic performance was reduced in patients with cough or movements during the cryoapplication. In these cases, the false positive rate increased, thus specificity declined to 31%. Even without any movements, the DMS specificity remained low, at 61%. Given this low diagnostic performance, the value of DMS in clinical practice should probably be debated.

### 4.2 CMAP threshold

We have confirmed the importance of respecting the 30% CMAP amplitude decrease threshold in order to obtain a complete recovery within the first 2 min. After this threshold, CMAP amplitude decreases fast; it takes a few seconds to drop under 50% (Figure 1). Here, we halted the cryoapplication 6.5 ± 4.2 s too late; as a consequence, the CMAP amplitude did not fully recover in 3 of 11 cases after 2 min. These results are in line with a previous publication from our team, and highlight the need for an automated CMAP analysis device (7).

### 4.3 DMS threshold

One could argue that the 30% threshold selection for the DMS is arbitrary. First, the DMS is available in clinical practice without any published data and no clear recommendation from the manufacturer about the cutoff. Thus, it was not possible to determine a relevant DMS cutoff to stop the cryoapplication. Second, because the sensitivity and the specificity are low, modification of the cutoff will not solve the low diagnostic performance. Finally, for lack of a better option, we decided to adopt the same cutoff as the CMAP. We determined the CMAP cutoff from an animal study. This 30% threshold was the best compromise from a statistical point of view. Later, in clinical studies, this threshold appeared to be particularly relevant, because if we are able to stop the cryoapplication before this point, CMAP amplitude fully recovers fast (7, 9, 11).

### 4.4 PNP rate

Phrenic nerve threatening was observed in 11/84 (13.1%) cryoapplications in 7/30 patients (23.3%). This value is relatively high, but has to be weighted by the low sample size of our study. One issue with PNP rate estimation in the literature is the lack of universal definition. PNP can be defined as a palsy happening at the time of the procedure, thus the reported rates are usually >10% (5, 9). PNP can also be defined as a persistent palsy at discharge, usually the day after the procedure; with this definition, reported PNP rates are usually <5% (3). The biggest study published to date is the YETI registry (17 356 patients), where the PNP rate was 4.2% (4). Thus PNP, even today, remains the most frequent complication of cryoballoon procedures. A complete recovery of phrenic nerve function could be achieved in 90% of patients (8). Nevertheless, these diaphragmatic function recoveries are usually based on normalization of the diaphragm position in x-ray images at rest. Relying on these static images could lead to underdiagnosis of permanent PNP (12).

In a recent study (6), the PNP rate was shown to be more important with Boston Scientific compared with Medtronic (Minneapolis, MN, USA) cryoballoons (15% vs. 7%; *P *= 0.048). One of the explanations put forward by the authors was DMS oversensitivity. Our results rather suggest a DMS lack of specificity.

### 4.5 Fatigue phenomenon

Muscular fatigue is defined as acute loss of contractile force after work. When sufficiently stressed, even the normal diaphragm can become fatigued (13). The major determinants of fatigue are the force and duration of diaphragmatic contraction (14). Supramaximal phrenic nerve pacing at 60 stimulations per minute for several minutes, as performed in cryoballoon procedures, generates a nonphysiologic vigorous diaphragmatic contraction. In 10/84 (11.9%) applications, a progressive decline in diaphragmatic movement, diagnosed by the DMS, was observed. Nevertheless, at the same time, no significant variation in CMAP was noticed. This pattern has already been described, particularly for the diaphragm (15). We were unable to highlight predictive factors for this phenomenon in our patients. Muscular fatigue represents an important limitation to the approach of phrenic nerve monitoring with diaphragmatic movements. Nevertheless, because the DMS has a low diagnostic value for PN threat, it cannot be excluded that these progressive decrease in the DMS value were only related to measurement errors.

### 4.6 Limitations

This was a monocentric study with a limited sample size and a single operator; these are limitations by nature.

## 5 Conclusion

Sensitivity and specificity of the DMS for the diagnosis of phrenic nerve threatening are low. The DMS gave an erroneous diagnosis in 52.4% of cryoapplications. Specificity was even lower in patients with movements at the time of the cryoapplications. If these results were to be confirmed by larger sample studies, the use of the DMS in clinical practice should be debated.

## Data Availability

The raw data supporting the conclusions of this article will be made available by the authors, without undue reservation.
